# Optimized Magnetic Stimulation Induced Hypoconnectivity Within the Executive Control Network Yields Cognition Improvements in Alzheimer’s Patients

**DOI:** 10.3389/fnagi.2022.847223

**Published:** 2022-03-15

**Authors:** Guixian Xiao, Yue Wu, Yibing Yan, Liying Gao, Zhi Geng, Bensheng Qiu, Shanshan Zhou, Gongjun Ji, Xingqi Wu, Panpan Hu, Kai Wang

**Affiliations:** ^1^Department of Neurology, The First Affiliated Hospital of Anhui Medical University, Hefei, China; ^2^The School of Mental Health and Psychological Sciences, Anhui Medical University, Hefei, China; ^3^Institute of Artificial Intelligence, Hefei Comprehensive National Science Center, Hefei, China; ^4^Anhui Province Key Laboratory of Cognition and Neuropsychiatric Disorders, Hefei, China; ^5^Collaborative Innovation Center for Neuropsychiatric Disorders and Mental Health, Hefei, China; ^6^Department of Neurology, Second People’s Hospital of Hefei City, The Hefei Affiliated Hospital of Anhui Medical University, Hefei, China; ^7^Center for Biomedical Imaging, University of Science and Technology of China, Hefei, China

**Keywords:** Alzheimer’s disease, executive control network, intermittent theta burst stimulation (iTBS), dorsolateral prefrontal cortex, cognitive function

## Abstract

Alzheimer’s disease (AD) is a severe neurodegenerative disease, which mainly manifests as memory and progressive cognitive impairment. At present, there is no method to prevent the progression of AD or cure it, and effective intervention methods are urgently needed. Network-targeted intermittent theta burst stimulation (iTBS) may be effective in alleviating the cognitive symptoms of patients with mild AD. The abnormal function of the dorsolateral prefrontal cortex (DLPFC) within executive control network (ECN) may be the pathogenesis of AD. Here, we verify the abnormality of the ECN in the native AD data set, and build the relevant brain network. In addition, we also recruited AD patients to verify the clinical effects of DLPFC-targeted intervention, and explor the neuro-mechanism. Sixty clinically diagnosed AD patients and 62 normal controls were recruited to explore the ECN abnormalities. In addition, the researchers recruited 20 AD patients to explore the efficacy of 14-session iTBS treatments for targeted DLPFC interventions. Functional magnetic resonance imaging and neuropsychological assessment of resting state were performed before and after the intervention. Calculate the changes in the functional connectivity of related brain regions in the ECN, as well as the correlation between the baseline functional connectivity and the clinical scoring scale, to clarify the mechanism of the response of iTBS treatment to treatment. Our results showed that compared with normal control samples, the brain function connection between the left DLPFC and the left IPL within the ECN of AD patients was significantly enhanced (*t* = 2.687, *p* = 0.008, FDR-corrected *p* = 0.045). And we found that iTBS stimulation significantly reduced the functional magnetic resonance imaging signal between the left DLPFC and the left IPL in the ECN (*t* = 4.271, *p* < 0.001, FDR-corrected *p* = 0.006), and it was related to the improvement of the patient’s clinical symptoms (*r* = −0.470, *p* = 0.042). This work provides new insights for targeted brain area interventions. By targeted adjusting the functional connection of ECN to improve the clinical symptoms and cognitive function of AD patients.

## Introduction

Alzheimer’s disease (AD) is a severe neurodegenerative disease that mainly manifests as memory and progressive cognitive impairment (Molteni and Rossetti, 2017). Data from the 2018 Annual Report of the World Alzheimer’s Disease (Prince and Matthew, 2018) show that there are approximately 50 million AD patients in the world at present, and the incidence continues to rise. AD has become the seventh cause of death in the world, and it is one of the most important health and social crises of the 21st century (Prince and Matthew, 2018). AD patients are mainly treated with drugs (Blennow et al., 2006), but these drug treatments can only delay the course of the disease to a certain extent, and they cause adverse reactions (Mielke et al., 2012). At present, there is no method to prevent the progression of AD or cure it (Godyn et al., 2016). With the advancement of science and technology, physical therapy methods have gradually been applied to the field of neurodegenerative diseases, such as transcranial magnetic stimulation (TMS) and transcranial electrical stimulation.

The abnormal function of the executive control network (ECN) may be the pathogenesis of AD (Dhanjal and Wise, 2014; Zhao et al., 2018, 2019). Studies have shown that the impaired response of the ECN during verbal memory contributes to the decline in memory performance (Dhanjal and Wise, 2014). Heterogeneity exists in previous studies, which may be related to different ECN construction methods (Shirer et al., 2012; Li et al., 2019). The research on ECN encompasses the dorsolateral prefrontal cortex (DLPFC) related to working memory and attention; the inferior parietal lobule (IPL) related to bottom-up attention and episodic memory; middle frontal gyrus (MFG) related to executive ability; and middle temporal gyrus (MTG) related to language function (Seeley et al., 2007; Vincent et al., 2008; Liu et al., 2021). Previous studies based on *a priori* selection of brain networks have shown that the functional connectivity between specific central executive networks and different areas of the brain is weakened (Dhanjal and Wise, 2014; Joo et al., 2016; Cai et al., 2017), which may explain the impairment and severity of cognitive function in AD patients (Chandra et al., 2019; Jones et al., 2019). Research on the functional connectivity of the resting state MRI of AD shows that DLPFC dysfunction in the early stage of AD manifests as memory impairment, especially the impairment of its executive components (Kaufman et al., 2010; Kumar et al., 2017; Puttaert et al., 2020). As an important node of the ECN (Seeley et al., 2007), understanding the functional mechanisms of the DLPFC and other brain areas of the ECN is important for designing effective interventions for patients with AD. At present, magnetic resonance imaging (TMS) can improve cognition by targeting the network through functional magnetic resonance imaging.

Targeted cortical-hippocampal network TMS stimulation in adults increases the functional connection between the cortex-hippocampal network area, which demonstrates their role in improving the associative memory function (Wang et al., 2014). A study on the effect of high-frequency TMS on network-targeted stimulation showed that it promoted the recall ability of participants to a greater extent (Nilakantan et al., 2019). Repetitive TMS (rTMS) is a non-invasive, green treatment that can stimulate brain nerves through the skull without attenuation. At present, TMS mainly treats diseases by adjusting the balance between excitement and inhibition of the brain in both directions in clinical practice; rTMS can generate excitatory postsynaptic potentials, resulting in abnormal excitement of the nerves in the stimulated part, while low-frequency stimulation is the opposite. TMS is generally considered to mainly affect the cerebral cortex, relying on cognitive circuits in the subcortex and other deep structures, such as the cingulate gyrus and hippocampus. The DLPFC is located in the lateral cerebral cortex, usually close to the stimulation area, which stimulates the cognitive function and clinical symptoms of DLPFC and is expected to benefit from TMS (Miniussi and Rossini, 2011; Brunoni and Vanderhasselt, 2014; Penolazzi et al., 2015; Cappon et al., 2016).

Cognition is a broad concept. Different cognitive functions are maintained by different brain networks and are affected by stimulation techniques to varying degrees. Whether intervention in the DLPFC of AD patients can compensate for the functional connection within the ECN, and the relevance of cognitive function and the ECN is still unclear. In this study, we mainly explored the effect of the iTBS on targeted DLPFC within the ECN in AD patients to determine whether they can improve memory function and clinical symptoms by regulating the activities of the frontal and parietal lobes. We predicted that left-DLPFC-targeted iTBS stimulation improves cognitive function clinical symptoms in AD. The improvement of clinical symptoms was related to the change of functional connections within the ECN.

## Materials and Methods

### Participants

This study mainly recruited AD patients aged 50–85 in the neurology clinic or ward of the First Affiliated Hospital of Anhui Medical University. All AD patients satisfying the diagnostic criteria for probable Alzheimer’s disease based on the National Institute of Neurological and Communicative Disorders and Stroke and the Alzheimer’s Disease and Related Disorders Association (NINCDS-ADRDA) criteria (McKhann et al., 1984, 2011). The clinical dementia rating (CDR) was between 0.5 and 2 points. Scored 10–27 on the Mini-Mental State Examination (MMSE). Treatment with donepezil at a steady dose for at least 3 months prior to iTBS until the treatment was completed. The exclusion criteria were as follows: patients with a history of drug abuse, alcoholism, or mental illness; patients with severe heart, lung, liver, and kidney dysfunction; acute and chronic infection; patients with craniocerebral trauma or severe cerebrovascular disease; and allergic constitution. For the normal controls (NCs), we recruited right-handed people who matched the AD group in age and gender. The exclusion criteria were participates without Alzheimer’s disease. The rest exclusion criteria for NCs were the same as those for AD patients.

The study was approved by the Anhui Medical University Ethics Committee, and all NCs and patients signed informed consent forms.

### Process

A total of 60 patients with AD were recruited during the first phase of the study; 62 age- and gender-matched NCs were also recruited. All participants were given a complete set of cognitive assessment scales by professional clinicians to assess their cognitive abilities and clinical symptoms. The resting state fMRI data were used to analyze the functional relationships between different parts of the ECN. In the second phase of the study we conducted an intervention study. A total of 20 AD patients were included. Each participant needed to be treated once a day for two consecutive weeks. After the first assessment and completion of the MRI scan, the resting motor threshold (RMT) was measured (Schutter and van Honk, 2006). During the completion of treatment, all patients received a stable dose of donepezil. All patients received complete neuropsychological tests and multimodal MRI scans before receiving iTBS treatment and within 24 h after the last stimulation treatment.

### Clinical Symptoms and Multi-Domain Cognition Assessments

The neuropsychology of the patients was assessed by a clinical investigator. The following neuropsychological test battery including the CDR, Global Deterioration Scale (GDS), Lawton-Brody Activities of Daily Living (ADL) scale, Montreal Cognitive Assessment (MoCA, Mandarin-version), Mini-mental State Examination (MMSE), AD8 scale and the Neuropsychiatric Inventory (NPI) were administered to evaluate whole cognitive function and clinical symptoms (Lezak et al., 2004). The CDR is a widely used multidimensional assessment of an individual’s internal cognitive, behavioral, and functional decline, based on the individual’s previously acquired abilities in these areas. The GDS is a set of staging methods developed by Reisberg et al. (1982) to assess the symptoms of AD. There were seven stages ranging from normal (no cognitive decline) to very severe cognitive decline. The scale was scored by interviewing patients and caregivers and was not objective. The higher the score, the more severe the symptoms. The CDR and GDS were used to grade the severity of AD and other dementias, which were widely used multidimensional assessment of an individual’s internal cognitive, behavioral, and functional decline, based on the individual’s previously acquired abilities in these areas (Reisberg et al., 1982; Morris, 1993). The ADL is mainly used to assess the daily living ability of the subjects. The AD8 is an assessment tool for rapid screening for cognitive abnormalities. The MMSE and MOCA were used to assess participants’ overall cognitive function. The NPI scale is used to assess the mental and behavioral symptoms of dementia patients. Based on the caregiver’s perception of the patient’s behavior and perceived distress. The Hachinski Ischemic Scale (HIS) was mainly used for the differential diagnosis of Alzheimer’s disease and vascular dementia. The severity of emotional symptoms in AD patients was evaluated by Hamilton Anxiety Scale and Hamilton Depression Scale. The Chinese version of the Auditory Verbal Learning Test (CAVLT, A/B), Clock-Drawing Test (CDT), Stroop test (Color/Word/Interference), Color Trail Test A/B (CTT A/B), Verbal Fluency Test (VFT) and Digital Span (forward/backward) were used to evaluate multiple cognitive domains. The Boston Naming Test (BNT) was used to investigate the ability of naming. Among them, CAVLT, Digital Span, and MMSE, A/B versions were adopted in the study and randomly used.

### Transcranial Magnetic Stimulation Intervention

#### Personalization and Image Navigation Transcranial Magnetic Stimulation

Individualized and image-navigated TMS uses individual 3D-T1 images to develop individualized targets for the stimulation for each participant. The stimulus target was defined as a sphere of the brain area of interest with a radius of 10 mm centered on the Montreal Neurology Institute (MNI) coordinates (−38, 44, 26) in standard magnetic resonance space (Mir-Moghtadaei et al., 2015; Wang et al., 2020; Wu et al., 2021). SPM^1^ were used to standardize and segment individualized magnetic resonance images and the TMS-target (Ji et al., 2017) to convert MNI coordinates to personalized TMS targets. The generated individualized stimulation targets and magnetic resonance images were all imported to the frameless navigation system (Visor 2.0). During stimulation, the same researcher placed the figure-eight MagStim coil under the navigation of Visor 2.0 and the connection point of the coil on the brain area of the stimulation target, which was a tangent to the skull, making the coil handle and the brain midline 45° angle oblique to the rear.

#### Intermittent θ Explosive Magnetic Stimulation

The parameters of iTBS were as follows: Resting Motor Threshold (RMT, more than 5 out of 10 consecutive stimuli can evoke the minimum motor evoked potential amplitude of the first dorsal extensor pollicis brevis muscle of >50 μV stimulus intensity) with a stimulation intensity of 70% (Schutter and van Honk, 2006). Each iTBS sequence released 600 pulses for a total of three sequences; each had an interval of 15 minutes and there were 1,800 pulse stimulations in total. For every 200 milliseconds, three pulses were continuously applied at a frequency of 50 Hz; each stimulation lasted for 2 s, with an interval of 8 s for rest, lasting for 192 s (Huang et al., 2005; Wang et al., 2020; Wu et al., 2020). The iTBS treatment was administered using a 70 mm air-cooled figure-of-eight coil and MagStim^2^ stimulator. To prevent hearing damage during treatment, the participants wore earplugs, closed their eyes during the process and at rest, and maintained a comfortable sitting posture. Before, during, and after the treatment, additional dialog between the participants and the researcher was avoided; the participants were asked if they had any symptoms of discomfort after the stimulation.

### Resting-State Functional Magnetic Resonance

#### Data Acquisition

The rs-fMRI image acquisition for all participants was mainly carried out at the Imaging Center of the University of Science and Technology of China, Hefei, Anhui Province. During the scan, all participants were asked not to fall asleep, close their eyes, not think about anything special, and keep their bodies still. A 3.0T MRI scanner (Discovery GE750w; GE Healthcare, Buckinghamshire, United Kingdom) composed of 217 echo plane imaging bodies was used for functional imaging. A total of 188 slices of T1-weighted anatomical images were acquired (voxel size = 1 mm × 1 mm × 1 mm; Repetition time = 8.16 ms; Echo time = 3.18 ms; flip angle = 12°; field of view = 256 mm × 256 mm; slice thickness = 1 mm). The parameters for rs-fMRI uses EPI scanning sequence were as follows (a total of 9,982 images were collected): Repetition time = 2,400 ms; slice thickness = 3 mm, Echo time = 30 ms, continuous slices = 46, voxel size = 3 mm × 3 mm × 3 mm), Flip angle = 90°, matrix size = 64 × 64, field of view = 192 mm × 192 mm.

#### rs-fMRI Preprocessing

We preprocess resting state functional magnetic resonance imaging data mainly using resting state functional magnetic resonance imaging toolkit (DPARSF) and SPM8^2^ (Chao-Gan and Yu-Feng, 2010). After deleting the first five volumes of data, the data was preprocessed, including slice time correction, head movement correction, spatial normalization, and smoothing. There was no significant difference in head movement between the two groups. And participants with a maximum displacement of 3 mm in any of the x, y, or z directions of head movement during the entire scan were excluded. We resampled the voxel size to 3 mm × 3 mm × 3 mm. Spatial normalization is performed using MNI’s standard EPI template. A 4 mm full-width half-maximum Gaussian filter is used for spatial smoothing. The removal of physiological high-frequency noise and low-frequency drift is performed by linear detrending and time bandpass (0.01–0.08 Hz) filtering (Cordes et al., 2001). In order to eliminate the influence of interference covariates, we perform linear regression on head movement parameters, global average signal, brain white matter signal, and cerebrospinal fluid signal (Fox et al., 2009).

#### Resting-State Functional Connection Analysis

**FIGURE 1:**
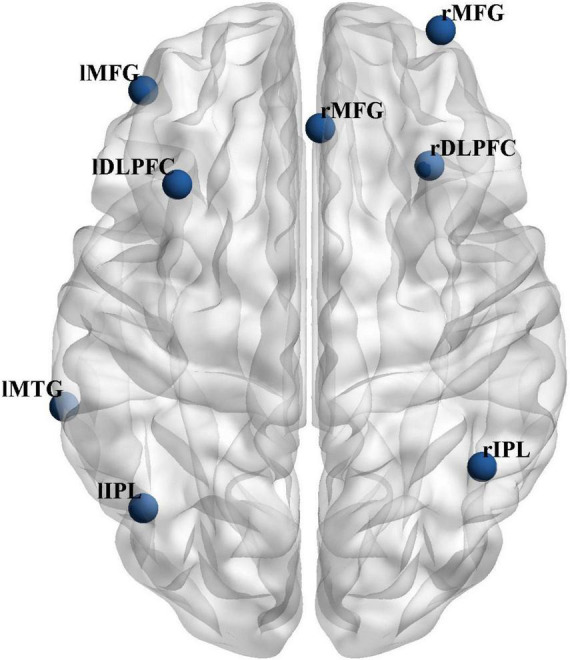
Location of the bilateral ECN seeds.

The locations of these subdivided seeds are shown in Figure 1. To explore the abnormal functional connections and clusters between the ECN structures, five bilateral seed points were selected from the ECN, based on previous studies (Fox et al., 2009) (see Supplementary Table 1). Create a spherical seed point with a radius of 3.5-mm around each seed point coordinate (Kennis et al., 2015). The BOLD time series of voxels between the seed regions are averaged to generate the reference time series of the seed region for each seed region.

### Statistical Analysis

The analysis was performed using SPSS23 (v 23.0, IBM, Armonk, NY, United States). Cross-sectional neuropsychological assessment data was statistically analyzed using the independent sample *t*-test, and the paired *t*-test was used to assess changes in neuropsychological assessment data before and after treatment. Pearson correlation analysis was used to do correlation analysis. The Fisher z-transform is used to normalize the correlation ROI signals. Individual z-signals was used for analysis. And the obtained test value was corrected using FDR. FDR correction is to correct each *p*-value and convert it to *q*-value. *q* = p × n/rank, where rank refers to the order in which *p*-values are sorted from the smallest to the largest. The probability value *p* < 0.05 (two-tailed) was considered to be statistically significant.

## Results

### Demographic and Clinical Information in Baseline

Participant demographic and clinical information are shown in Table 1. The average age and education (±standard deviation) in years were 64.508 (±9.005) for AD/63.984 (±7.623) for NCs and 8.783 (±4.694) for AD/9.540 (±4.099) for NCs, respectively. Their Hakinski Ischemic Scale (HIS) scores were 1.117 ± 0.691; all were less than 4. There were no significant differences in gender, age, and education between the AD and control groups. In the AD group, the MMSE, MoCA, NPI, ADL, CDR, and GDS were worse than those of the same age group. According to the multidimensional cognitive assessment, the AD group performed significantly worse on tests that assessed memory (immediate recall, delayed recall, and recognition), sub-tests that assessed information processing (DS-F/B, Trail Making Test A/B, and Stroop interference Test), and tests that assessed language function (VFT) in Table 1.

**TABLE 1:** The demographic and clinical characteristics and neuropsychological test results of the Alzheimer’s Patients and normal controls in the cross-sectional study.

Variable	Alzheimer’s patients [*n* = 60, means (SD)]	Healthy controls [textitn = 62, means (SD)]	χ ^2^/t	*p*-value
**Demographic**				
Gender (M/F)	26/34	26/36	0.024	0.876
Age (years)	64.508(9.005)	63.984(7.623)	0.350	0.727
Education (years)	8.783(4.694)	9.540(4.099)	–0.959	0.342
**Clinical characteristics**				
HIS	1.117(0.691)	0.968(0.567)	1.305	0.194
NPI-frequency * severity	11.086(12.9110)	0.063(0.504)	**6.498**	<0.0001****
NPI-distress	4.069(5.057)	0.016(0.126)	**6.102**	<0.0001****
ADL	31.103(11.243)	20.175(0.555)	**7.395**	<0.0001****
CDR	1.026(0.581)	0.071(0.176)	**12.020**	<0.0001****
GDS	3.828(0.704)	1.317(0.715)	**19.438**	<0.0001****
**Neuropsychological tests**				
AD8	5.071(2.100)	0.952(1.223)	**11.479**	<0.0001****
MMSE	18.000(6.250)	28.873(1.601)	**13.174**	<0.0001****
MoCA	11.852(5.822)	25.444(3.605)	**15.572**	<0.0001****
HAMA	4.733(4.153)	2.937(3.636)	**2.556**	0.012*
HAMD	3.696(3.421)	2.952(3.381)	1.155	0.250
CDT	1.650(1.219)	3.902(0.436)	**–13.386**	<0.0001****
**Memory**				
CAVLT - immediately	2.521(1.644)	8.851(1.775)	**–20.581**	<0.0001****
CAVLT - delay	0.590(1.203)	9.778(2.661)	**–24.907**	<0.0001****
CAVLT - recognition	10.295(4.112)	14.317(1.045)	**–7.411**	<0.0001****
**Attention**				
digit span test(forward)	5.818(1.634)	6.968(1.576)	**–3.887**	<0.0002***
digit span test(backward)	3.127(1.187)	4.333(1.426)	**–5.013**	<0.0001****
**Executive function**				
Stroop Color test	42.825(35.050)	21.537(6.402)	**4.197**	<0.0001****
Stroop Word test	69.120(59.354)	30.304(10.147)	**4.526**	<0.0001****
Stroop Interference test	99.169(83.708)	20.425(16.741)	**6.483**	<0.0001****
Trail Making A	199.429(137.609)	70.482(23.389)	**6.008**	<0.0001****
Trail Making B	372.821(245.119)	128.731(47.240)	**7.266**	<0.0001****
**Linguistic function**				
VFT - animal	9.839(3.296)	17.206(3.802)	**–11.226**	<0.0001****
BNT	16.426(4.817)	26.377(4.499)	**–11.051**	<0.0001****

*M, male; F, female; HIS, Hachinski Ischemic Scale; NPI, Neuropsychiatric Inventory; NA, not applicable; ADL, Lawton-Brody Activities of Daily Living; CDR, Clinical Dementia Rating; GDS, Global Deterioration Scale, MMSE, Mini-mental State Examination; MoCA, Montreal Cognitive Assessment Test; HAMA, Hamilton Anxiety Rating Scale; HAMD, Hamilton Depression Rating Scale; CDT, Clock Drawing Test; CAVLT, Chinese version of the Auditory Verbal Learning Test; VFT, Verbal Fluency Test; BNT, Boston Naming Test; ^a^: Chi-squared test; ^b^: Mann-Whitney U test; ^c^: Independent sample T test.*

*p < 0.05; **p < 0.01; ***p < 0.001; ****p < 0.0001.

### Group Difference Within Executive Control Network in Baseline

**FIGURE 2:**
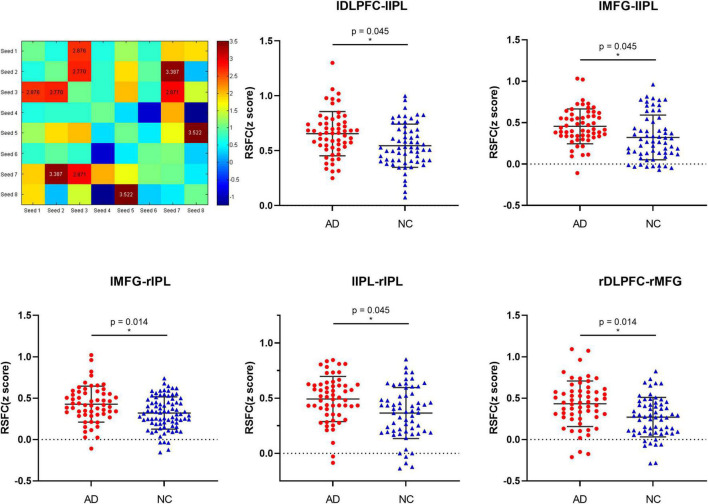
The differences in resting-state functional connectivity (mean *Z*-value) within the bilateral ECN seeds between the AD patients and normal controls.

The results of the seed point RSFC within the network between groups show that in the ECN, compared with the NCs, the functional connections in the network between left DLPFC and left IPL (*t* = 2.687, *p* = 0.008, FDR-corrected *p* = 0.045), left MFG and left IPL (*t* = 2.770, *p* = 0.007, FDR-corrected *p* = 0.045), left MFG and right IPL (*t* = 3.387, *p* = 0.001, FDR-corrected *p* = 0.012), left IPL and right IPL (*t* = 2.871, *p* = 0.005, FDR-corrected *p* = 0.045), right DLPFC and right MFG (*t* = 3.522, *p* = 0.001, FDR-corrected *p* = 0.014) was significantly increased in AD patients (see Figure 2 and Supplementary Table 2). There was no significant difference in the functional connectivity of the resting state within the remaining network in the groups.

### Correlation Analysis of Resting-State Functional Connection Within the Executive Control Network in Two Groups

The correlation analysis of RSFC within the bilateral seeds in the ECN showed that the strength of the functional connection in the ECN was significantly correlated with cognition, respectively. In AD group, correlation analysis shows that the functional connection strength between the left DLPFC and the right DLPFC was negatively correlated to VFT (*r* = −0.272, *p* = 0.046), and positively correlated with AD8 (*r* = 463, *p* = 0.003), GDS (*r* = 0.323, *p* = 0.015), and CTT-B (*r* = 0.350, *p* = 0.039). Correlation analysis in NC group shows that the functional connection strength between the left DLPFC and the left IPL is related to CAVLT-immediate (*r* = −0.303, *p* = 0.017), CAVLT-delay (*r* = −0.310, *p* = 0.014) and CTT-B (*r* = −0.271, *p* = 0.045) is negatively correlated. The analysis of the remaining cognition in each group was shown in Table 2.

**TABLE 2:** Pearson correlation analysis of RSFC within the ECN after iTBS stimulation in AD patients and normal controls.

Alzheimer’s patients (*n* = 60)				Normal controls (*n* = 62)			
Seed	Neuropsycholo-gical test	*R*	*p*-value	Seed	Neuropsycholo-gical test	*R*	*p*-value
Seed1-Seed5	AD8	0.463	0.003**	Seed1-Seed3	CAVLT- immediately	–0.303	0.017*
	GDS	0.323	0.015*		CAVLT-delay	–0.310	0.014*
	VFT	–0.272	0.046*		CTT-B	–0.271	0.045*
	CTT-B	0.350	0.039*	Seed1-Seed5	Stroop Color test	0.307	0.016*
Seed1-Seed6	HAMD	0.296	0.030*	Seed1-Seed6	CDT	–0.281	0.030*
Seed1-Seed8	CTT-B	0.524	0.001**	Seed1-Seed7	GDS	0.280	0.028*
Seed2-Seed6	CDT	–0.360	0.006**		CTT-B	0.294	0.029*
	HAMA	0.296	0.024*	Seed1-Seed8	CDT	–0.274	0.034*
	HAMD	0.389	0.004**	Seed2-Seed3	CTT-B	–0.280	0.038*
	NPI-frequency*severity	0.336	0.012*	Seed2-Seed5	VFT	0.258	0.199*
	VFT	–0.411	0.003**	Seed2-Seed6	NPI-frequency*severity	–0.315	0.013*
Seed3-Seed4	HAMA	0.294	0.025*		NPI-distress	–0.315	0.013*
	HAMD	0.301	0.027*	Seed3-Seed4	Stroop Word test	0.312	0.014*
	ADL	0.272	0.045*	Seed3-Seed5	Stroop Color test	0.256	0.047*
	DS(backward)	–0.274	0.047*	Seed3-Seed7	MMSE	0.300	0.018*
	BNT	–0.460	0.001**	Seed4-Seed7	AD8	0.279	0.028*
Seed3-Seed6	HAMA	0.276	0.036*		Stroop Word test	0.282	0.028*
	HAMD	0.378	0.005**	Seed4-Seed8	Stroop Word test	0.253	0.049*
Seed4-Seed5	DS(backward)	–0.292	0.034*	Seed6-Seed7	NPI-frequency*severity	–0.325	0.01*
Seed4-Seed6	HAMD	0.326	0.016*		NPI-distress	–0.325	0.01*
	NPI-frequency*severity	0.278	0.040*		Stroop Color test	0.307	0.016*
Seed5-Seed8	AD8	0.367	0.020*		Stroop Word test	0.344	0.007**
	VFT	–0.303	0.026*	Seed6-Seed8	Stroop Color test	0.298	0.02*
Seed7-Seed8	MMSE	0.336	0.010**		Stroop Word test	0.270	0.035*
	MoCA	0.270	0.040*	Seed7-Seed8	HAMD	–0.263	0.039*
	CAVLT-delay	0.285	0.030*				

*NPI, Neuropsychiatric Inventory; NA, not applicable; ADL, Lawton-Brody Activities of Daily Living; GDS, Global Deterioration Scale, MMSE, Mini-mental State Examination; MoCA, Montreal Cognitive Assessment Test; HAMA, Hamilton Anxiety Rating Scale; HAMD, Hamilton Depression Rating Scale; CDT, Clock Drawing Test; CAVLT, Chinese version of the Auditory Verbal Learning Test; CTT, Color Trail Test; VFT, Verbal Fluency Test; DS, Digit Span test; BNT, Boston Naming Test.*

*p < 0.05; **p < 0.01.

### Improvement of Clinical Symptoms Within the Executive Control Network of Alzheimer’s Disease Patients After Intermittent Theta Burst Stimulation

It can be found that iTBS can significantly improve the overall cognitive function, daily function, mental behavioral symptoms, and multiple cognitive functions of AD patients (*P* < 0.05). The results show that iTBS can significantly improve the overall cognitive function of AD patients, including MoCA (*t* = −5.229, *p* < 0.001), MMSE (*t* = −5.3449, *p* < 0.001), CAVLT-immediately (*t* = −4.129, *p* < 0.001), CAVLT-delay (*t* = −4.129, *p* < 0.001), CAVLT-recognition (*t* = −3.256, *p* = 0.004) and BNT (*t* = −3.685, *p* = 0.002) significantly improved after stimulation. In addition, both the family members and caregivers of AD patients found a certain degree of improvement after iTBS treatment, which was reflected in the score of the NPI-frequency × severity (*t* = 3.427, *p* = 0.003) and NPI-distress (*t* = 3.209, *p* = 0.005), as described in Table 3.

**TABLE 3:** Improvement clinical characteristics and cognitive function after iTBS treatment in AD patients.

Variable	pre [*n* = 19, means (SD)]	post [*n* = 19, means (SD)]	*t*	*p*-value
**Clinical characteristics**				
NPI-frequency*severity	8.05(10.54)	3.16(5.81)	3.427	0.003**
NPI-distress	2.47(2.91)	1.31(1.91)	3.209	0.005**
ADL	29.10(8.59)	27.47(8.20)	3.759	0.001**
CDR	0.87(0.46)	0.81(0.48)	1.455	0.163
GDS	3.89(0.74)	3.63(0.76)	2.041	0.056
**Neuropsychological tests**				
MMSE	19.26(4.73)	21.58(5.75)	–5.349	< 0.0001****
MoCA	12.94(5.53)	15.68(6.53)	–5.229	< 0.0001****
HAMA	4.53(3.18)	2.84(2.31)	2.191	0.042*
HAMD	3.89(3.62)	1.53(2.17)	3.316	0.004**
CDT-4	1.42(0.84)	2.37(1.06)	–4.869	< 0.0001***
**Memory**				
CAVLT-immediately	2.69(2.18)	4.13(2.65)	–4.129	0.001**
CAVLT-delay	1.79(2.80)	3.53(3.93)	–3.124	0.006**
CAVLT-recognition	11.68(2.43)	13.05(2.15)	–3.256	0.004**
**Attention**				
digit span test(forward)	5.58(1.89)	6.21(1.68)	–2.721	0.014*
digit span test(backward)	3.26(1.59)	3.79(1.31)	–3.293	0.004**
**Executive function**				
Stroop Color test	44.38(28.51)	37.42(23.94)	2.304	0.034*
Stroop Word test	63.30(36.46)	54.57(31.32)	2.947	0.009**
Stroop Interference test	91.27(53.58)	81.28(50.48)	1.442	0.168
Trail Making A	245.95(180.38)	229.89(152.80)	1.430	0.170
Trail Making B	414.49(279.86)	399.52(268.05)	1.121	0.279
**Linguistic function**				
VFT-animal	9.47(4.13)	10.79(4.53)	–1.492	0.153
BNT	16.95(6.25)	19.58(5.98)	–3.685	0.002**

*M, male; F, female; HIS, Hachinski Ischemic Scale; NPI, Neuropsychiatric Inventory; NA, not applicable; ADL, Lawton-Brody Activities of Daily Living; CDR, Clinical Dementia Rating; GDS, Global Deterioration Scale, MMSE, Mini-mental State Examination; MoCA, Montreal Cognitive Assessment Test; HAMA, Hamilton Anxiety Rating Scale; HAMD, Hamilton Depression Rating Scale; CDT, Clock Drawing Test; CAVLT, Chinese version of the Auditory Verbal Learning Test; VFT, Verbal Fluency Test; BNT, Boston Naming Test.*

*p < 0.05; **p < 0.01; ***p < 0.001; ****p < 0.0001.

### The Resting-State Functional Connection Changes Within the Executive Control Network and Correlation Analysis After Intermittent Theta Burst Stimulation

**FIGURE 3:**
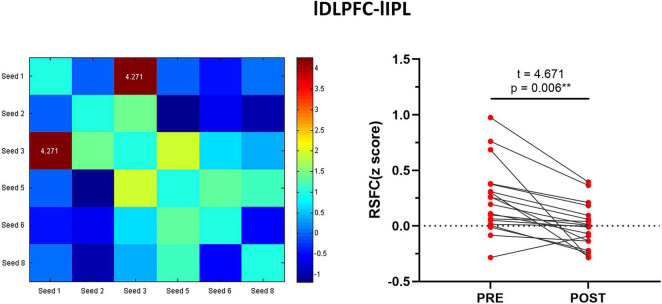
Differences before and after iTBS treatment in the Alzheimer’s Patients in the ECN.

The cognitive function assessment and rs-fMRI imaging studies of a total of 20 patients were included in the study. One patient had a large head movement and was deleted from the analysis. Finally, the data of 19 patients were included in the analysis. The seed point-whole brain RSFC analysis showed that the strength of the functional connection between the left DLPFC and the left IPL (*t* = 4.271, *p* < 0.001, FDR-corrected *p* = 0.006) decreased significantly after treatment (see Figure 3 and Supplementary Table 3). Correlation analysis showed that the improvement of GDS after iTBS stimulation was correlated with the changes in lDLPFC-lIPL strength in the ECN (*r* = −0.470, *p* = 0.042).

## Discussion

Our study found a significant cognitive decline in AD patients compared with normal controls, and RSFC analysis found enhanced functional connectivity of various seed points within the ECN, which was associated with cognitive function. Longitudinal analysis showed a significant increase in the MMSE, MoCA scores of patients with AD after 2 weeks of treatment. The improvements in the overall clinical neuropsychiatric symptoms, severity, and ability to live daily were observed during the pre-post phase. Cognitive performance across multi-domains improved significantly, especially on tests related to memory (CAVLT, MoCA, and VFT) and attention. In addition, this study revealed the underlying neural mechanism for iTBS related to improvement in the clinical symptoms of AD. This improvement was achieved by changing the functional connection between the dorsolateral prefrontal lobe and the subparietal lobules in the executive control network. However, this study had no significant effect on CDR and GDS grading, the executive function (including Stroop Interference test and Trail Making A/B) and verbal fluent test.

The overall cognitive function of AD patients, including memory function, attention, executive function and language function, orientation function, computing function, and visual-spatial function, was impaired, which was consistent with the reports of previous studies (Binetti et al., 1996; Baudic et al., 2006; Hodges, 2006; Kirova et al., 2015). RSFC analysis showed that the functional connectivity of each network node in the ECN was abnormal. Compared with normal controls, the functional connectivities of the left DLPFC and left IPL; left IPL and left MFG; left IPL and right IPL; left IPL and right IPL; and right DLPFC and right MFG were enhanced in AD patients. This abnormal pattern of enhanced functional connectivity may be caused by compensatory functional effects in the brain of AD patients (Crosson et al., 2013). Compared with healthy controls, AD patients had impairments of memory, attention, and executive function, and the abnormality of functional connectivity was related to some cognitive subdomains, which may be related to the adjustment of the working memory, attention, thinking, logical reasoning, and other advanced cognitive functions (Zhao et al., 2005; Lantrip et al., 2017). Previous studies also found that the cross-network functional connection of the triple network model (ECN, default mode network, salience network) of AD patients was significantly impaired, and the interaction of the triple-network model was impaired, which may have led to the decline of cognitive function (Li et al., 2019).

The iTBS can significantly improve the overall cognitive function, the ability of daily living, and mental behavior symptoms of AD patients. This result is consistent with the reports of previous studies (Ahmed et al., 2012; Balconi and Ferrari, 2012; Chou et al., 2020). This may be because targeted left-DLPFC iTBS stimulation may have increased the excitability and function. The abnormality of the ECN of AD patients may be related to its function (Li et al., 2019). TMS is usually used to understand the function of a single brain area, but this ignores the fact that TMS may affect the network through network nodes (Cameron et al., 2020). Studies have found that TMS targeting of the left DLPFC can improve memories of young people and patients with healthy aging and affect network functions (Vidal-Pineiro et al., 2014; Wang et al., 2014; Nilakantan et al., 2017; Kim et al., 2018). As the core node of the executive control network (Seeley et al., 2007), our study showed that targeted stimulation of the left DLPFC may further affect the excitability of the ECN. The change of functional connectivity between the left DLPFC and left IPL within the ECN after stimulation was correlated with the improvement of GDS, which indicated that correcting the abnormalities in the executive control network may be related to the improvement of cognitive function (Dhanjal and Wise, 2014). A possible neurological explanation for the improvement of clinical symptoms in AD patients is that iTBS is designed to counteract maladaptive neuroplasticity and promote adaptive changes. Decreased activity in these areas may be due to iTBS-induced neural stimulants that show more efficient or less laborious processing at different levels (Koechlin and Summerfield, 2007). This hypothesis is consistent with the neural efficiency hypothesis, which holds that individuals with improved cognitive ability have lower cortical metabolic rates (Kar and Wright, 2014). This is supported by previous literature, which has shown that high-frequency iTBS of the left DLPFC may be a useful supplement for the treatment of AD.

There are still several deficiencies in this study. First, the sample size was small. Second, this study was carried out only based on the effectiveness results of previous studies without setting up a control group, which may be mixed with the practice effect. Therefore, future studies with a larger sample size and longer intervention duration and placebo control group are needed to further verify the conclusion of this study. The stimulus targets in this study were based on the conventional therapeutic targets used in our laboratory in the past (Wang et al., 2020; Wu et al., 2021). In future treatments, we can use the abnormally linked brain regions of the ECN obtained from the study as stimulus targets. In addition, future studies could use electroencephalogram techniques combined with MRI to further explore the relationship between changes in neural brain networks and behavioral outcomes during TMS, especially the effect of baseline neural activity characteristics on intervention outcomes.

In summary, this study suggests that the improvement of psychobehavioral symptoms and cognitive dysfunction in AD patients may be related to the changes of RSFC in different brain regions of bilateral ECN. Our study provided preliminary findings that pinpoint the effect of iTBS for the left DLPFC on the clinical symptoms and RSFC between the lDLPFC and lIPL of the ECN of AD patients. This discovery will facilitate the development of effective targeted-left DLPFC non-invasive interventions for the cognitive rehabilitation of patients with AD.

## Data Availability Statement

The raw data supporting the conclusions of this article will be made available by the authors, without undue reservation.

## Ethics Statement

The studies involving human participants were reviewed and approved by the Anhui Medical University Ethics Committee, and all NCs and patients signed informed consent forms. The patients/participants provided their written informed consent to participate in this study. Written informed consent was obtained from the individual(s) for the publication of any potentially identifiable images or data included in this article.

## Author Contributions

All authors contributed significantly to, and approved, the content of this manuscript.

## Conflict of Interest

The authors declare that the research was conducted in the absence of any commercial or financial relationships that could be construed as a potential conflict of interest.

## Publisher’s Note

All claims expressed in this article are solely those of the authors and do not necessarily represent those of their affiliated organizations, or those of the publisher, the editors and the reviewers. Any product that may be evaluated in this article, or claim that may be made by its manufacturer, is not guaranteed or endorsed by the publisher.

## Footnotes

1
https://www.fil.ion.ucl.ac.uk/spm/software/spm8/

2
www.fil.ion.ucl.ac.uk/spm/software/spm8
