# Supplementary material for: Optimized Magnetic Stimulation Induced Hypoconnectivity Within the Executive Control Network Yields Cognition Improvements in Alzheimer’s Patients
Source: Front Aging Neurosci. 2022 Mar 15;14:847223. doi: 10.3389/fnagi.2022.847223 (PMC8965584; doi:10.3389/fnagi.2022.847223)
Supplement: Supplementary file 1 [file Table_1.docx]

| S1. Coordinates for the ten bilateral seeds are given in coordinates defined in Montreal Neurological Institute space. | | | | | |
| --- | --- | --- | --- | --- | --- |
| Seed | ACC region | BA | MNI coordinates | | |
|  |  |  | x | y | z |
|  | **Left ECN** |  |  |  |  |
| Seed 1 | Dorsolateral Prefrontal Cortex | 8 | -35 | 21 | 53 |
| Seed 2 | Middle Frontal Gyrus | 46 | -44 | 46 | -1 |
| Seed 3 | Inferior Parietal Louble | 39 | -44 | -65 | 44 |
| Seed 4 | Middle Temporal Gyrus | 20 | -65 | -38 | -12 |
|  | **Right ECN** |  |  |  |  |
| Seed 5 | Dorsolateral Prefrontal Cortex | 9 | 32 | 26 | 44 |
| Seed 6 | Middle Frontal Gyrus | 10 | 35 | 62 | 7 |
| Seed 7 | Inferior Parietal Louble | 40 | 46 | -54 | 49 |
| Seed 8 | Middle Frontal Gyrus | 8 | 3 | 36 | 44 |
| Abbreviations: BA, Brodmann area; MNI, Montreal Neurological Institute. ECN, Executive control network; The labels are the tag corresponding to the brain area in figures. | | | | | |
