# Supplementary material for: Optimized Magnetic Stimulation Induced Hypoconnectivity Within the Executive Control Network Yields Cognition Improvements in Alzheimer’s Patients
Source: Front Aging Neurosci. 2022 Mar 15;14:847223. doi: 10.3389/fnagi.2022.847223 (PMC8965584; doi:10.3389/fnagi.2022.847223)
Supplement: Supplementary file 2 [file Table_2.docx]

| S2. The t-value of the peaks within the clusters of significant different resting state functional connectivity between the Alzheimer’s patients and normal controls per seed in ECN (t-test). | | | | | |
| --- | --- | --- | --- | --- | --- |
| Seed | Alzheimer’s patients  (N=60, means±SD) | Healthy controls  (N=62, means±SD) | *t* | *p*-value | FDR-corrected  *p*-value |
| **Left ECN** |  |  |  |  |  |
| Seed1-Seed2 | 0.38(0.25) | 0.35(0.28) | 0.681 | 0.497 | 0.643 |
| Seed1-Seed3 | 0.64(0.21) | 0.55(0.20) | ***2.687*** | ***0.008***** | ***0.045**** |
| Seed1-Seed4 | 0.43(0.27) | 0.40(0.22) | 0.669 | 0.505 | 0.643 |
| Seed1-Seed5 | 0.36(0.22) | 0.31(0.20) | 1.233 | 0.220 | 0.440 |
| Seed1-Seed6 | 0.24(0.23) | 0.21(0.22) | 0.704 | 0.483 | 0.643 |
| Seed1-Seed7 | 0.34(0.22) | 0.25(0.23) | 1.922 | 0.057 | 0.199 |
| Seed1-Seed8 | 0.36(0.25) | 0.29(0.18) | 1.826 | 0.071 | 0.199 |
| Seed2-Seed3 | 0.44(0.22) | 0.32(0.27) | ***2.770*** | ***0.007***** | ***0.045**** |
| Seed2-Seed4 | 0.39(0.25) | 0.38(0.25) | 0.750 | 0.940 | 0.940 |
| Seed2-Seed5 | 0.21(0.24) | 0.13(0.22) | 1.820 | 0.071 | 0.199 |
| Seed2-Seed6 | 0.41(0.20) | 0.38(0.20) | 0.986 | 0.326 | 0.571 |
| Seed2-Seed7 | 0.42(0.20) | 0.31(0.19) | ***3.387*** | ***0.001***** | ***0.014**** |
| Seed2-Seed8 | 0.39(0.22) | 0.38(0.21) | 0.236 | 0.814 | 0.844 |
| Seed3-Seed4 | 0.51(0.27) | 0.48(0.25) | 0.728 | 0.468 | 0.643 |
| Seed3-Seed5 | 0.36(0.20) | 0.29(0.19) | 2.020 | 0.046 | 0.184 |
| Seed3-Seed6 | 0.30(0.23) | 0.27(0.22) | 0.756 | 0.451 | 0.643 |
| Seed3-Seed7 | 0.48(0.22) | 0.36(0.23) | ***2.871*** | ***0.005***** | ***0.045**** |
| Seed3-Seed8 | 0.33(0.22) | 0.27(0.21) | 1.467 | 0.145 | 0.338 |
| Seed4-Seed5 | 0.25(0.23) | 0.24(0.19) | 0.389 | 0.698 | 0.782 |
| Seed4-Seed6 | 0.32(0.22) | 0.35(0.19) | -0.901 | 0.370 | 0.609 |
| Seed4-Seed7 | 0.39(0.22) | 0.31(0.19) | 2.107 | 0.037 | 0.173 |
| Seed4-Seed8 | 0.28(0.25) | 0.33(0.16) | -1.235 | 0.220 | 0.440 |
| Seed5-Seed6 | 0.26(0.25) | 0.24(0.26) | 0.455 | 0.650 | 0.776 |
| Seed5-Seed7 | 0.37(0.25) | 0.30(0.20) | 1.651 | 0.101 | 0.257 |
| Seed5-Seed8 | 0.43(0.27) | 0.27(0.24) | ***3.522*** | ***0.001***** | ***0.014**** |
| Seed6-Seed7 | 0.45(0.26) | 0.40(0.22) | 1.094 | 0.276 | 0.515 |
| Seed6-Seed8 | 0.37(0.26) | 0.35(0.22) | 0.305 | 0.761 | 0.819 |
| Seed7-Seed8 | 0.44(0.26) | 0.42(0.20) | 0.435 | 0.665 | 0.776 |
| Abbreviations: BA, Brodmann area; MNI, Montreal Neurological Institute. ECN, Executive control network; The labels are the tag corresponding to the brain area in figures.  * p < 0.05; **p < 0.01. | | | | | |
