# Supplementary material for: Optimized Magnetic Stimulation Induced Hypoconnectivity Within the Executive Control Network Yields Cognition Improvements in Alzheimer’s Patients
Source: Front Aging Neurosci. 2022 Mar 15;14:847223. doi: 10.3389/fnagi.2022.847223 (PMC8965584; doi:10.3389/fnagi.2022.847223)
Supplement: Supplementary file 3 [file Table_3.docx]

| S3. The t-values of resting state functional connectivity before and after iTBS treatment in the Alzheimer’s Patients per seed in ECN. | | | | | |
| --- | --- | --- | --- | --- | --- |
| Seed | Pre  (N=19, means±SD) | Post  (N=19, means±SD) | t | p-value | FDR-corrected  p-value |
| Seed1-Seed2 | 0.15(0.21) | 0.16(0.21) | -0.043 | 0.966 | 0.993 |
| Seed1-Seed3 | 0.23(0.30) | -0.01(0.19) | ***4.271*** | ***<0.001****** | ***0.006***** |
| Seed1-Seed5 | 0.25(0.23) | 0.25(0.23) | -0.009 | 0.993 | 0.993 |
| Seed1-Seed6 | 0.03(0.17) | 0.06(0.27) | -0.421 | 0.679 | 0.849 |
| Seed1-Seed8 | 0.27(0.23) | 0.26(0.26) | 0.043 | 0.966 | 0.993 |
| Seed2-Seed3 | 0.12(0.22) | 0.02(0.22) | *1.384* | 0.183 | 0.653 |
| Seed2-Seed5 | 0.16(0.17) | 0.22(0.23) | -1.180 | 0.253 | 0.653 |
| Seed2-Seed6 | 0.22(0.18) | 0.26(0.25) | -0.609 | 0.550 | 0.849 |
| Seed2-Seed8 | 0.16(0.20) | 0.20(0.21) | -0.980 | 0.339 | 0.726 |
| Seed3-Seed5 | 0.08(0.13) | 0.01(0.14) | **1.910** | **0.071** | 0.533 |
| Seed3-Seed6 | 0.05(0.17) | 0.02(0.16) | 0.627 | 0.538 | 0.849 |
| Seed3-Seed8 | 0.05(0.17) | 0.02(0.17) | 0.425 | 0.675 | 0.849 |
| Seed5-Seed6 | 0.31(0.33) | 0.21(0.28) | 1.303 | 0.208 | 0.653 |
| Seed5-Seed8 | 0.29(0.31) | 0.21(0.24) | *1.158* | 0.261 | 0.653 |
| Seed6-Seed8 | 0.14(0.26) | 0.17(0.29) | -0.568 | 0.576 | 0.849 |
| Abbreviations: BA, Brodmann area; MNI, Montreal Neurological Institute. ECN, Executive control network; The labels are the tag corresponding to the brain area in figures.  * p < 0.05; **p < 0.01; ***p < 0.001 | | | | | |
